# Balancing orbital volume reduction and redistribution for a tailored surgical treatment in Graves’ ophthalmopathy

**DOI:** 10.1007/s00417-020-04807-4

**Published:** 2020-06-25

**Authors:** Victor Vlad Costan, Constantin-Catalin Ciocan-Pendefunda, Mihai Liviu Ciofu, Otilia Boisteanu, Daniel Vasile Timofte, Liliana Gheorghe, Camelia Bogdanici, Cristina Preda

**Affiliations:** 1 Department of Oral and Maxillofacial Surgery, Grigore T. Popa University of Medicine and Pharmacy, Universitatii Str, no. 16, 700115 Iasi, Romania; 2 Klinik für Kiefer- und Gesichtschirurgie, Universitätsklinikum Schleswig-Holstein, Campus Lübeck, Ratzeburger Allee 160, 23538 Lübeck, Germany; 3 Department of Oral and Maxillofacial Surgery, Anesthesiology, Grigore T. Popa University of Medicine and Pharmacy, Universitatii Str, no. 16, 700115 Iasi, Romania; 4 Department of Surgery, Grigore T. Popa University of Medicine and Pharmacy, Universitatii Str, no. 16, 700115 Iasi, Romania; 5 Department of Radiology, Grigore T. Popa University of Medicine and Pharmacy, Universitatii Str, no. 16, 700115 Iasi, Romania; 6 Department of Ophthalmology, Grigore T. Popa University of Medicine and Pharmacy, Universitatii Str, no. 16, 700115 Iasi, Romania; 7 Department of Endocrinology, Grigore T. Popa University of Medicine and Pharmacy, Universitatii Str, no. 16, 700115 Iasi, Romania

**Keywords:** Graves’ ophthalmopathy, Proptosis, Exophthalmos, Decompression, Orbit, Diplopia

## Abstract

**Aim:**

The purpose of this study is to share our experience on the use of different orbital decompression techniques, as well as the principles followed for deciding the most case-appropriate procedure that ensured the most favorable outcomes.

**Methods:**

We reviewed the Graves’ ophthalmopathy cases operated over the course of 14 years, regarding the presenting signs, the imaging evaluation, the degree of exophthalmos, the type of surgical orbital decompression performed, and the postoperative outcomes.

**Results:**

All 42 patients identified presented with proptosis, with 92.8% cases of bilateral proptosis. The main addressing concern was functional in 54.8% cases and aesthetic in 45.2% patients. CT was used for the preoperative evaluation in all cases. In total, 81 orbits were operated. The orbital decompression surgery involved only the orbital fat in 7.4% of orbits and associated fat and bone decompression in the other 92.6% of orbits. The postoperative results were favorable in all cases regarding both appearance and function, with minimal postoperative complications.

**Conclusion:**

The adequate selection of the most suitable procedure based on the characteristics of each case is the prerequisite for a successful surgery. We found that the association of fat and bone decompression of various extents is most permissive in tailoring the degree of decompression to the existing requirements.

## Introduction

Almost half of patients with Graves’ disease develop ophthalmopathy [1, 2]. Their life quality is greatly impacted by the developing orbital and periorbital changes. The associated exophthalmos, double vision, retraction of the eyelids, periorbital edema, strabismus, and optic neuropathy determine aesthetic and functional disturbances to various degrees. Addressing these modifications is compelling in order to improve the psycho-social life of the patients.

Surgery follows an initial unsuccessful medical treatment and is usually indicated in the inactive stage of the disease. It is estimated that almost one-fifth of Graves’ ophthalmopathy patients will undergo surgical treatment over a 10-year period [1]. Surgical treatment is aimed at decreasing or redistributing the enlarged orbital volume by performing either a fat removal orbital decompression, a bone removal decompression, or an association of the two. Although the principle remains the same, there are many variations regarding the choice of the procedure, the access, the amount of tissue to be removed, and the necessity for concomitant or subsequent procedures for the amelioration of the periorbital changes associated with proptosis. The preference for either one of the existing methods is based on the characteristics of each case and the experience of the surgeon. Constant attempts are made at improving the existing techniques for achieving better outcomes regarding appearance and function, while reducing the incidence of complications.

The purpose of this study is to present our experience on the surgical treatment of Graves’ ophthalmopathy and to discuss the technical details that proved most advantageous in achieving favorable postoperative outcomes.

## Material and methods

We conducted a retrospective review of Graves’ ophthalmopathy patients that underwent orbital decompression surgery between January 2006 and March 2019. We included patients with a follow-up period of minimum 6 months. We excluded cases that underwent emergency decompression surgery for the presence of optic neuropathy, as well as patients with previous orbital surgery performed for a different condition.

Information was gathered concerning the presenting signs and symptoms, the degree of exophthalmos, previous treatments, imaging investigations, the surgical technique, the postoperative outcomes, and additional procedures.

## Results

### Patients

A total of 42 cases were identified, of which 31 (73.8%) women and 11 (26.2%) men, with a 1:2.8 male to female ratio. The age of the patients was between 18 and 71. All patients presented with proptosis, showing globe projection measurements between 22 and 31 mm. Globe projection was measured clinically during the ophthalmologic evaluation, by using Hertel’s exophthalmometer, and confirmed by measurements performed on the CT (computed tomography) images. There were 39 (92.8%) cases presenting with bilateral proptosis and 3 (7.2%) cases of unilateral exophthalmos. From the bilateral proptosis group, 21 cases had asymmetric exophthalmos, with a difference greater than 1.5 mm between the two eyes. Diplopia was reported by 12 (28.6%) of the included patients. Other described symptoms were the presence of pain in 14 (33.3%) cases, retro-ocular tension sensation in 12 (28.6%) patients, a subjective decrease in visual field perception in 13 (30.1%) cases, and blurred vision in 11 (26.2%) patients.

Although all patients had proptosis upon clinical examination, only 19 (45.2%) of them presented for the aesthetic changes associated with exophthalmos, while the other 23 (54.8%) of the patients reported one or several of the associated symptoms as main concerns, including diplopia, pain, retro-ocular tension, changes in visual field perception, and blurred vision.

All the included patients were evaluated using a preoperative CT scan. Routine preoperative endocrinologic and ophthalmologic examinations were performed in all cases, evaluating the thyroid status, measuring the degree of exophthalmos, and determining the visual acuity, the presence of double vision, and the associated changes of the periorbital tissues and the eye movements. The indication for surgery was established in a multidisciplinary team comprising of an endocrinologist, an ophthalmologist, a radiologist, and a maxillofacial surgeon.

### Surgical procedure

The surgical decompression was indicated in the chronic stage of the disease, subsequent to achieving a normal thyroid function for at least 6 months, and when the previously attempted medical orbital decompression was unsuccessful. Treatment by orbital irradiation had been attempted before surgery in 4 of the patients.

A bilateral orbital decompression was performed in 39 (92.8%) patients, while in 3 (7.2%) cases, the procedure was unilateral. In total, 81 orbits were operated. In six (7.4%) orbits, decompression surgery involved only the removal of orbital fat. For the other 75 (92.6%) orbits, an associated fat and bony wall decompression was performed, consisting of a one-wall decompression in 31 orbits (38.3%) and a two-wall decompression in 44 orbits (54.3%).

Orbital fat decompression alone or in association with bony decompression involved the removal of fat from all four quadrants in all cases, but in different amounts, considering the individual variations as determined by clinical and imaging examinations. In 28 (34.6%) orbits, only the extraconal fat was addressed, while in 53 (65.4%) orbits, both the extraconal and the intraconal fat compartments were accessed. The amount of fat removed varied between 0.1 and 3.5 ml for the superior quadrants, including the intraconal fat, and between 0.5 and 2.9 mL for the inferior quadrants. To access the superior fat quadrants, a superior eyelid blepharoplasty type of skin incision was performed. For accessing the inferior orbit, we used an inferior eyelid transpalpebral incision in 32 (39.5%) orbits and a transconjunctival incision for 49 (60.5%) orbits.

In 75 (92.6%) orbits, a bone decompression was associated to the fat decompression. In 31 orbits (38.3%), the inferior orbital wall was addressed. In the other 44 (54.3%) orbits, the inferior and the medial orbital walls were involved, but only the bone situated medially from the infraorbital canal was removed. The inferior orbital wall was accessed through an osteotomy in the inferior orbital rim in 18 (22.2%) orbits, followed by the repositioning of the sectioned rim segment. In the other cases, the bone removal from the orbital floor was performed without a rim osteotomy. In all cases, the first step of the procedure was the removal of orbital fat from all quadrants, followed by performing the bone decompression.

In 56 (69.1%) orbits, a levator palpebrae aponeurosis sectioning was performed at the time of orbital fat decompression, for lengthening of the eyelid. In 12 (28.6%) of the included cases, botulinum toxin injections were administered postoperatively for the persistent superior eyelid retraction after orbital decompression. In five (11.9%) cases, an eyelid skin excess was found after decompression surgery, involving the superior eyelids in two patients, the inferior eyelids in one case, and both the superior and the inferior eyelids in other two patients. A subsequent blepharoplasty was performed for those patients. There was only one (2.4%) patient diagnosed with unilateral postoperative retraction of the inferior eyelid, in which a palatal mucosal graft was performed 1 year and a half after decompression surgery.

### Postoperative care

Cold packs were applied in the first 3 days after surgery, consisting of intermittent application of compresses soaked in cold saline over the eyes. Sleeping with the head of the bed elevated was recommended.

Anti-inflammatory eye drops were prescribed for 5 days after surgery. Intravenous corticoid treatment was administered to all patients for 7 days after surgery. Antibiotic treatment was administered for 5 days after the decompression procedure.

The visual function and postoperative pain were closely monitored in the first hours following surgery in order to early detect the development of an expanding retrobulbar hematoma. An ophthalmologic evaluation was performed the day after and 7 days postoperative to evaluate the visual acuity, the status of diplopia, and the range of eye movements.

For accurately evaluating the proptosis reduction after the complete healing of the orbital tissues, measurements of proptosis using Hertel’s exophthalmometer were performed and documented 6 months following decompression surgery. The follow-up period was minimum 6 months and maximum 13 years.

### Postoperative outcomes

Proptosis was significantly reduced in all patients following surgery. Measurements with Hertel’s exophthalmometer performed 6 months postoperative showed a decrease in proptosis of minimum 1 mm and maximum 6.4 mm, compared with the preoperative measurements. In the patients that underwent an isolated orbital fat decompression, the minimum proptosis reduction was 1 mm and the maximum was 4.1 mm. In the group of patients with fat and one-wall bone decompression, the proptosis reduction was minimum 2.3 mm and maximum 4.9 mm. The mixed orbital fat and two-wall orbital decompression group showed a decrease in proptosis of minimum 3.2 mm and maximum 6.4 mm. From the 21 patients with asymmetric proptosis, symmetry was achieved after surgery in 19 cases.

The most common complications immediately after surgery were chemosis in 17 (40.5%) cases, important periorbital tissue edema in 18 (42.8%) patients, and widespread periorbital ecchymosis in 11 (26.2%) cases that subsided in between 5 and 14 days after surgery. In 2 (4.8%) patients, a minor superior eyelid hematoma developed in the first day after surgery that did not necessitate drainage. We did not encounter any compressive retrobulbar hematoma development in the immediate postoperative period. There were no recorded infections.

Preoperative diplopia that was present in 12 cases disappeared 2 weeks after surgery in 9 patients. The other 3 patients in which double vision persisted after surgery were directed for further strabismus surgery. For the patients without complaints of preoperative double vision, a transient diplopia developed after the procedure, but gradually disappeared within 1 and 2 weeks following orbital decompression surgery. The additional complaints consisting of orbital pain, tension, sensation of visual field decrease, and blurred vision were improved postoperative.

Infraorbital nerve hypoesthesia was present after surgery in all patients in which the decompression involved the orbital floor bone situated both medially and laterally from the infraorbital canal, in which the nerve was freed from its bony canal. For those patients, the hypoesthesia gradually disappeared within 2 months, with two exceptions when it persisted for more than 12 months. In the cases in which a partial removal of the orbital floor was performed, involving the area located medially from the infraorbital canal, there were no complaints of decreased sensation in the territory of the infraorbital nerve after surgery.

**Fig. 1:**
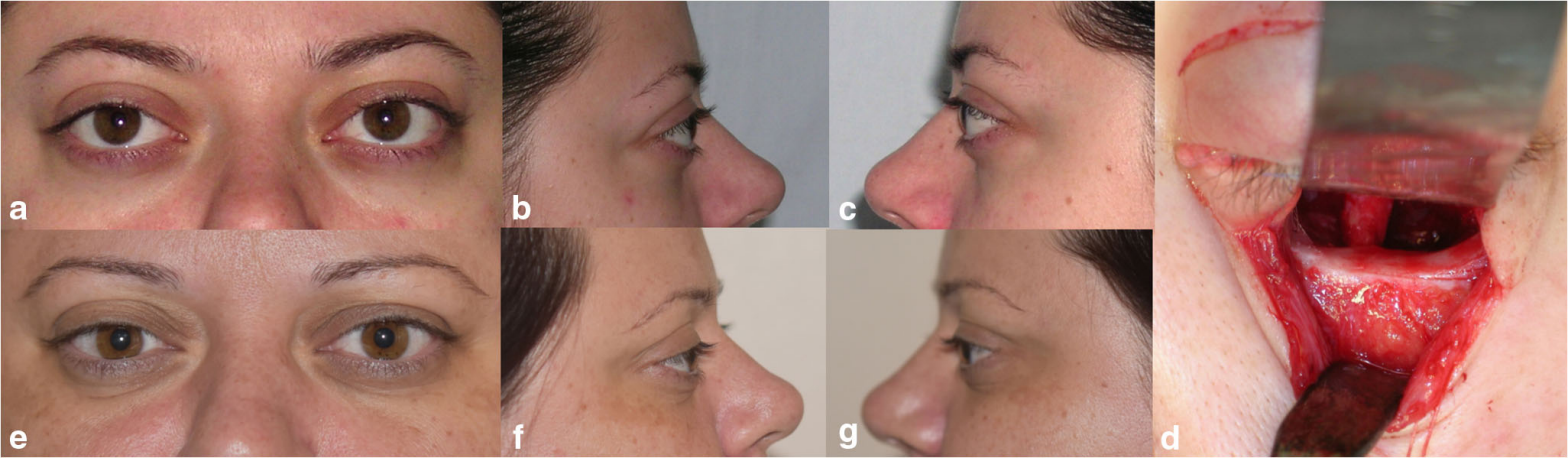
Case 1: Graves’ ophthalmopathy patient with bilateral proptosis in which a bilateral orbital decompression of the orbital fat and of the orbital floor was performed. **a** Preoperative frontal view. **b** Preoperative right profile view. **c** Preoperative left profile view. **d** Intraoperative aspect showing the bony decompression of the left orbital floor and the presence of the freed infraorbital nerve. **e** Postoperative frontal view of the patient 12 years after surgery showing the stable resolution of proptosis. **f** Right profile view 12 years after surgery. **g** Left profile view 12 years postoperative

**Fig. 2:**
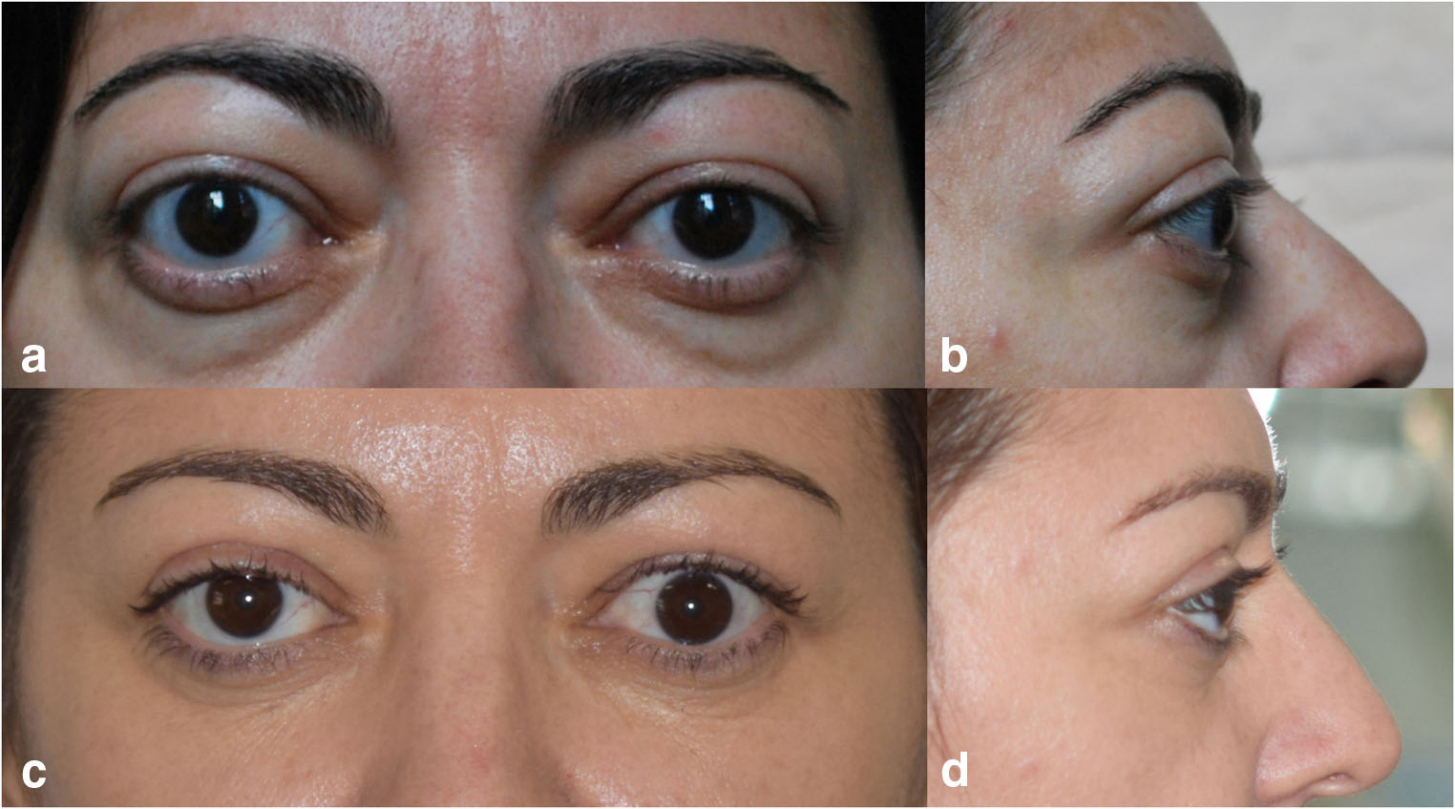
Case 2: Graves’ ophthalmopathy patient with bilateral asymmetric proptosis in which a mixed fat and bony decompression of the right orbit and isolated fat decompression of the left orbit were performed. **a** Preoperative frontal view. **b** Preoperative right profile view. **c** Postoperative frontal view of the patient, 8 years after orbital decompression surgery, showing the restoration of symmetry and resolution of diplopia. **d** Postoperative right profile view of the patient, 8 years after orbital decompression surgery

**Fig. 3:**
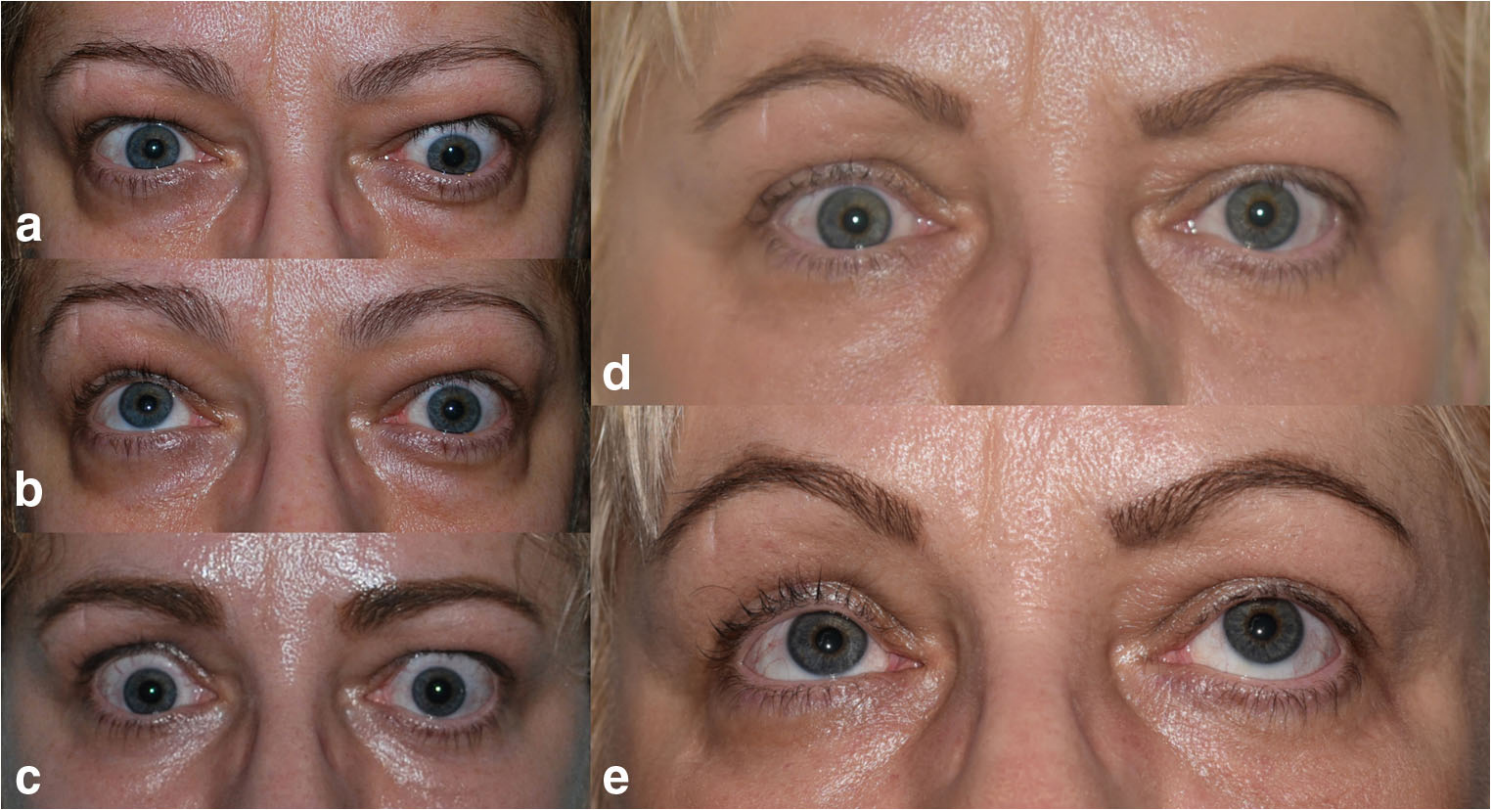
Case 3: Preoperative aspect of a Graves’ ophthalmopathy patient in which diplopia was the main concern. A mixed fat and bony decompression surgery was performed. **a** Preoperative frontal view during forward gaze, showing the unequal level of the globes. **b** Preoperative aspect of the patient during upward gaze showing the movement restriction in the left eye. **c** Frontal view of the patient 2 years after bilateral orbital decompression surgery showing the equal level of the globes, as well as the presence of bilateral superior eyelid retraction. **d** Frontal view of the patient following the injection of botulinum toxin performed for the superior eyelid retraction, showing normal eyelid position that was stable 10 years after the injection. **e** Frontal view of the patient during upward gaze, 12 years after surgery, demonstrating the absence of movement restriction

The favorable postoperative outcomes regarding proptosis reduction are exemplified by images of three clinical cases (Figs. 1, 2, and 3).

## Discussion

In conformity with the treatment guidelines consensually established by the European Group on Graves’ Orbitopathy (EUGOGO) [3], we performed surgical orbital decompression in patients with significant complaints, in which the disease had been inactive for minimum 6 months. Most surgeons follow these criteria for determining who should undergo decompression surgery, but there is great variability in the choice of techniques employed, considering the characteristics of each case, as well as the experience and specialization of the surgeons involved.

In our opinion, the most important step in determining the type of decompression surgery that is indicated for each case is the imaging evaluation, which offers an accurate assessment of the most important anatomical structures that are known to be involved in the development of Graves’ ophthalmopathy—the orbital fat and the extraocular muscles. In addition to providing important details for treatment selection, imaging is important for excluding the presence of inflammatory conditions of the paranasal sinuses, such as ethmoid and maxillary sinusitis, which constitute contraindications for the removal of the corresponding bony walls that separate the orbital cavity from the involved sinus cavity. Particularly for unilateral exophthalmos, CT is required for excluding an intraorbital tumor growth originating in the orbital tissues or in the neighboring structures with subsequent orbital invasion and globe protrusion [4].

In our study, the imaging technique of choice for patient evaluation was CT. A recent review [5] states that almost 96 % of surgeons are in favor of CT evaluation, while only 3% opt for MRI (magnetic resonance imaging). Braun et al. [6] also advocate for the use of CT in the preoperative planning, claiming that the investigation can be useful in anticipating the amount of proptosis reduction that can be achieved surgically. In our study, CT images were carefully analyzed preoperatively for establishing the amount of proptosis, the symmetry, and the predominance of orbital fat or muscle volume growth. The distribution of the fat was evaluated by analyzing the volumes of the extraconal and intraconal compartments. This step in the preoperative planning allowed the selection of the appropriate technique, as well as approximating the type and extent of tissue removal. The removal of more than one bony wall could thus be avoided in the patients demonstrating important fat volume enlargement.

Using CT scans, Ugradar and Rootman [7] have found that prevailing orbital fat expansion is more frequent in younger Graves’ orbitopathy patients, while muscle hypertrophy could not be related to a certain age group. We did not find such a correlation between age and the amount of orbital fat predominance. In our case series, consecutive to analyzing the orbital volume changes using the CT images, we found few patients with a prevalence of orbital fat enlargement in which an isolated orbital fat removal decompression was performed. In most cases, due to the observation of a more balanced fat and muscle volume growth, we opted for an associated orbital fat and osseous decompression surgery, with variations in the choice of access, number, and degree of orbital wall removal. This technique rendered optimal results regarding proptosis reduction and diplopia resolution.

Fat decompression alone is supported by some authors due to the stated increased incidence of new onset diplopia associated with bone decompression surgery, with the possible explanation of changing the axis of the globes [1, 4]. Such findings are not congruent with the results of our study, since we did not encounter any cases of persistent new onset diplopia after surgery. Additionally, we observed good results regarding the resolution of preexisting diplopia following combined fat and bone decompression surgery. Olivari [8] considered that removing a large amount of orbital adipose tissue would lead to better functional results, in the detriment of aesthetics, due to the resulting appearance of “sunken eyes.” In our experience, this cosmetic disadvantage of fat decompression can be managed by association of a simultaneous osseous expansion and thus decreasing the amount of fat excision. The inconveniences of each separate technique are therefore reduced. The combined technique allows a more efficient individual treatment adaptation and customization in accordance to the anatomical modifications of the fat and muscle compartments, particularly important for asymmetric cases. By coupling the two decompression methods, we achieved significant proptosis reduction with good functional results and symmetry improvement.

Regarding the choice of orbital walls for bone decompression, we prefer addressing the inferior and medial walls due to the convenient access, easy bone removal, and the favorable outcomes achieved. An advantage to this technique is the transconjunctival approach that offers a simple access to both the inferior and the medial orbital walls with no visible scars. Similarly, a survey performed in 2015 outlined that most of the included surgeons opted for a two-wall orbital decompression, with more than half of them addressing the inferior and medial walls, while the rest preferred medial and lateral wall expansion [9].

Some authors support the removal of the lateral orbital wall alone or in conjunction with other walls for more important proptosis. Although good results are reported by some studies [5, 10, 11], the lateral orbital wall is quite thick and its removal more technically challenging [12]. Furthermore, there are studies describing complications such as temporal hollowing and oscillopsia following lateral wall decompression surgery [13, 14]. Ugradar and Rootman stated that less orbital volume expansion is achieved by lateral wall removal when compared with medial wall decompression [7]. This may be due to the presence of the temporalis muscle behind the lateral orbital wall, while the sinuses found on the other side of the inferior and medial orbital walls offer more space for decompression.

Jefferis et al. [11] underlined the advantage of performing the removal of the medial wall and posterior aspect of the inferior wall for creating space for the optic nerve in optic neuropathy cases. We consider that this additional space can also be achieved after the removal of intraconal fat with consecutive quick improvement of the visual function in those patients.

Dutton [12] observed that the medial and the inferior rectus muscles are the ones most frequently found enlarged in Graves’ orbitopathy. These muscles are favorably situated from an anatomical and surgical viewpoint, since they are in proximity to the thin medial and inferior orbital walls that can easily be accessed and removed to allow space for the enlarged muscle volume. The previously mentioned advantages of medial and inferior wall decompression explain the favorable outcomes obtained in our study by using this orbital expansion technique.

Concerns were raised by authors regarding the possible postoperative onset of double vision following inferior wall bony decompression due to the prolapse of the inferior rectus muscle into the maxillary sinus and altering of the vertical globe level [15]. However, many factors must be considered such as the extent of bony decompression and the bilaterality of the procedure, as well as the inflammatory modifications of the extraocular muscles. In our study, there were no patients with persistent postoperative diplopia. Nevertheless, from the patients with preexisting preoperative diplopia, three continued to complain of double vision despite the performed orbital decompression. They were further directed for strabismus surgery due to the finding of asymmetric enlargement of the extraocular muscles in the context of Graves’ disease. In this regard, Wang et al. [16] argued that inflammatory changes including myositis and fibrosis are the likely cause of the asymmetric expansion of the extraocular muscles, most often involving the inferior and the medial rectus muscles, resulting in restrictive strabismus.

Naik et al. [17] argued that each orbital wall removed leads to approximately 2-mm proptosis reduction. Braun et al. [6] states that removing orbital fat increases the reduction of exophthalmos by an additional 2–4 mm, particularly when orbital fat expansion is predominant. Clauser et al. [18] stated that an association of fat and osseous decompression is most appropriate for proptosis greater than 26 mm, in the presence of both adipose tissue and muscle volume expansion. The benefits of associating bone and fat removal for optimal proptosis reduction are also outlined by the favorable results of our study. Similar outcomes using the combined technique are reported by other authors [19, 20].

A known inconvenience associated with osseous decompression involving the orbital floor is the possible occurrence of infraorbital nerve hypoesthesia. Unlike our findings of only transient nerve impairment, some studies report a rate between 1 and 25% permanent infraorbital hypoesthesia, corresponding to different techniques [21]. Attempts to decrease the onset of this complication were made by technique refinements. Clauser et al. performed an osteotomy surrounding the nerve foramen in order to free the nerve and decrease pressure [22]. Our experience suggests that this type of approach to the orbital floor by rim osteotomy results in more cases of postoperative infraorbital sensory impairment, although transient. Another technique variation showed improved results in our hands, regarding the reduced occurrence of infraorbital nerve hypoesthesia. It involves a more balanced bone decompression, by removing the medial orbital wall and only the internal part of the orbital floor, instead of the entire surface. Other authors also support this technique of osseous orbital decompression [23]. By the partial removal of the inferior orbital wall, the dissection and injury of the nerve is avoided. Additionally, there is less weight on the nerve, due to the remaining protection offered by the bony canal. Another advantage is avoiding the need for a cutaneous incision, since the medial and inferior walls can be easily accessed through a transconjunctival approach if a rim osteotomy is not intended [24].

Another issue to be addressed in Graves’ orbitopathy patients is the hypertrophy or hyperactivity of the levator palpebrae muscle, which can lead to eyelid retraction and the consecutive exacerbation of the ocular discomfort [12]. Our approach involved the severing of the levator aponeurosis during decompression surgery if intraoperative evidence of muscle hypertrophy was found. In twelve patients with remaining superior eyelid retraction after decompression surgery, botulinum toxin injections were performed into the same muscle resulting in sufficient eyelid lengthening. Contrarily, we found an eyelid excess after orbital decompression surgery in five of the included patients that further necessitated blepharoplasty. A false palpebral retraction could be the reason for this occurrence, involving an enlarged eyelid that is draped over the exophthalmic eye and revealed only after the decompression surgery, when the globe returns to its normal position in relation to the orbital margins. The occurrence of skin excess in some of the patients with Graves’ exophthalmia may be due to the slow progressive stretching of the eyelids over the proptotic globe during the course of the disease [25].

Our study revealed a relatively low complication rate, without any major ones. This is consistent with the findings of other authors describing only minor complications associated to the transpalpebral or endoscopic techniques [11]. Severe complications, like cerebrospinal fluid leaks and impaired visual acuity, were reported by some authors in relation with classic transnasal procedures, performed without a visual endoscopic control [11, 21].

Ultimately, the goal of decompression surgery is to increase the patient’s quality of life by improving the aesthetics and functionality, while decreasing subjective complaints. Most of the existing studies describe a decrease in orbital discomfort following orbital decompression surgery [26, 27]. In our study, all the included patients described a reduction in local pain and retro-ocular pressure feeling.

## Conclusion

Our experience regarding orbital decompression surgery for Graves’ orbitopathy demonstrated that with proper preoperative planning and careful selection of the type of procedure, it is possible to ensure enough proptosis correction and the resolution of diplopia, while reducing complications. A technique that proved most useful in the majority of our cases was the association of fat and osseous decompression in various amounts that were individually adapted, in an attempt to maximize the benefits and reduce the downfalls of each separate procedure.
